# Chylous ascites occurring after low anterior resection of the rectum successfully treated with an oral fat-free elemental diet (Elental^®^)

**DOI:** 10.1007/s12328-012-0304-7

**Published:** 2012-05-13

**Authors:** Gakuryu Nakayama, Daisuke Morioka, Takashi Murakami, Hideki Takakura, Yasuhiko Miura, Shinji Togo

**Affiliations:** Department of Surgery, Yokohama Ekisaikai Hospital, 1-2 Yamada-cho, Naka-ku, Yokohama, 231-0036 Japan

**Keywords:** Chylous ascites, Colorectal surgery, Elemental diet

## Abstract

Chylous ascites occurring after abdominal surgery is rare. Despite being potentially critical, there is no definite treatment guideline because of its rarity. Here we present a case of massive chylous ascites occurring after rectal surgery which was successfully treated with an oral fat-free elemental diet (ED). A 67-year-old man underwent low anterior resection with para-aortic lymphadenectomy for advanced rectal cancer. Early postoperative course was uneventful and the patient was discharged from hospital 10 days after surgery; however, after discharge, abdominal distension rapidly developed. Abdominal computed tomography (CT) performed 3 weeks after surgery revealed massive ascites and laboratory findings showed remarkable hypoproteinemia and lymphopenia. Urgent diagnostic paracentesis showed the ascites to be a white milky fluid containing high levels of triglycerides (564 mg/dl), leading to a diagnosis of chyloperitoneum. Daily nutrition of the patient was entirely with a fat-free ED (30 kcal/kg/day of Elental^®^, Ajinomoto Pharmaceutical Co. Ltd, Tokyo, Japan). After the initiation of oral Elental^®^, abdominal distension, hypoproteinemia, and lymphopenia gradually improved. Abdominal CT performed 7 weeks after surgery showed no ascitic fluid in the abdomen, and thereafter a normal diet was initiated. Since then, no relapse of chyloperitoneum has been proven. As a result, the chylous ascites was successfully treated in the outpatient clinic.

## Introduction

Chylous ascites occurring after abdominal surgery is rare but has been reported in certain populations [1–4]. The reported incidence of this condition has varied because it is rare and sometimes asymptomatic [1–4]. If it becomes symptomatic, however, a massive loss of fat/protein-enriched fluid and lymphocytes can cause severe malnutrition and susceptibility to infection, leading to fatality [1–7]. Because of its rare occurrence, there is no consensus for management of this potentially lethal complication [1–7]. Therefore, reporting of new cases is useful for sharing valid experiences.

Sporadic case reports showed that total parenteral nutrition (TPN) with or without octreotide (or somatostatin) was effective for eradicating this condition [3–7]. However, TPN management requires hospitalization and a central venous catheter which is an invasive procedure [8]. Furthermore, treatment with octreotide and/or somatostatin requires close medical vigilance and high medical costs [8–11]. Here we present a case of massive chylous ascites occurring after rectal surgery which was successfully treated with an oral fat-free elemental diet (ED) in the outpatient clinic.

## Case report

**Fig. 1:**
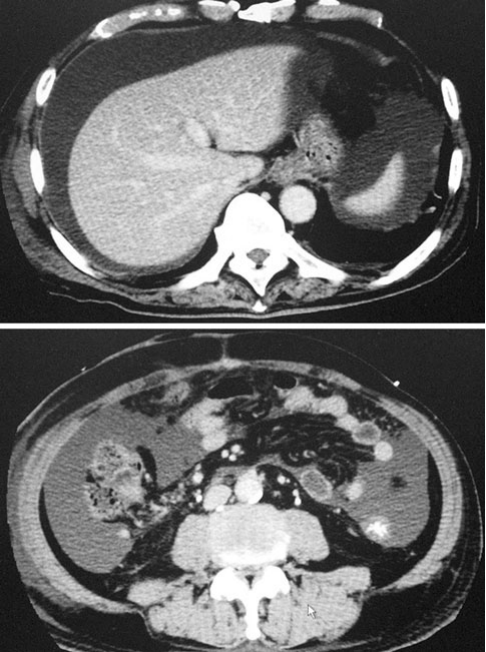
Abdominal computed tomography performed 3 weeks after surgery. Massive ascites was seen in the abdomen

**Fig. 2:**
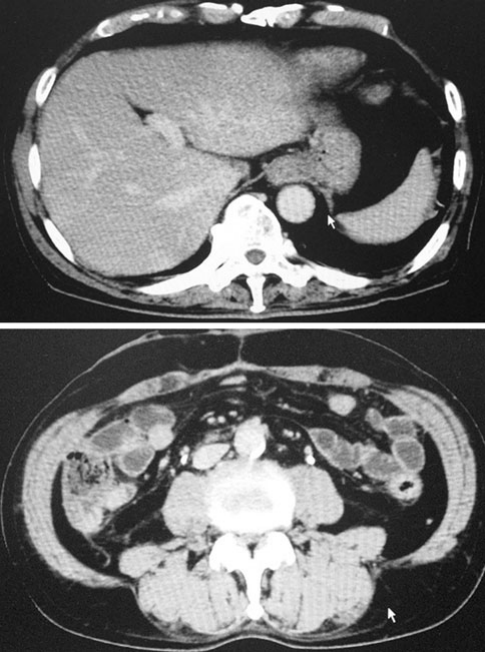
Abdominal computed tomography performed 7 weeks after the surgery. No ascitic fluid was seen in the abdomen

A 67-year-old man underwent low anterior resection with para-aortic lymphadenectomy for advanced rectal cancer. Neither neoadjuvant radiotherapy nor chemotherapy was performed. Early postoperative course was uneventful and the patient was discharged from hospital 10 days after the surgery. After discharge, however, abdominal distension developed and rapidly deteriorated. On the routine outpatient visit planned 21 days after surgery, the patient suffered from a severe sense of abdominal fullness and remarkable abdominal distension was noted with 92 cm of abdominal girth measured at the umbilicus. An emergency abdominal computed tomography (CT) scan proved that abdominal distension was caused by massive ascites (Fig. 1). Urgent diagnostic paracentesis showed that the ascites was a white milky fluid containing high levels of triglycerides (546 mg/dl), leading to a diagnosis of massive chylous ascites. Simultaneous laboratory findings showed severe hypoproteinemia (total protein 4.5 g/dl, albumin 2.1 g/dl) and lymphopenia (white cell count 3000/μl, lymphocyte count 150/μl). However, neither abdominal pain nor other signs suggestive of infection were found. Based on sporadic evidence that TPN with or without octreotide was reported to be effective for eradicating chyloperitoneum occurring after abdominal surgery [1–7], emergency admission was offered to the patient but rejected due to social reasons. Therefore, an oral fat-free ED (30 kcal/kg/day of Elental^®^, Ajinomoto Pharmaceutical Co. Ltd, Tokyo, Japan) was initiated. Elental^®^ was given orally two or three times a day. Oral intake was strictly restricted to Elental^®^, water, and sugar/milk-free beverage. The patient was seen weekly in the outpatient clinic. After the initiation of oral Elental^®^, abdominal distension gradually improved. Most notably, the sense of abdominal fullness dramatically abated a few days after starting Elental^®^. His abdominal girth decreased to 82 cm and an abdominal CT scan showed no ascitic fluid in the abdomen 42 days after surgery. At that time, hypoproteinemia and lymphopenia improved (total protein 7.2 g/dl, albumin 4.2 g/dl, white cell count 6300/μl, lymphocyte count 2010/μl). Accordingly, the ED was totally replaced by a normal diet the following day, after which no abdominal distension was noted. As a result, the chylous ascites was successfully treated in the outpatient clinic. Six months after rectal surgery, the patient underwent partial hepatectomy (S8 segmentectomy) for liver metastasis originating from the rectal cancer. At that time, no ascitic fluid was seen in the abdomen. The patient is doing well without evidence of cancer recurrence or chyloperitoneum at 18 months after the initial rectal surgery (Fig. 2).

## Discussion

Chylous ascites is caused by the unrecognized disruption of a major lymphatic channel during lymphadenectomy [1–7]. The most important issue regarding this rare but significant complication relates to prevention. Meticulous and systematic use of ligation or hemoclips before dividing distinguishable lymphatic vessels is essential for preventing this condition, notably in areas such as the para-aortic region where abdominal lymphatic systems converge and lymphatic fistula is apt to occur [1–7]. However, lymphatic vessels are often indistinguishable and thus chylous ascites cannot be completely prevented. Therefore, it is very important to establish a treatment strategy for this complication.

The choice of treatment for chylous ascites after abdominal surgery ranges from surgery to conservative treatment [1–7]. If the origin of the leak is definitely detected then surgical repair is preferred [12, 13]. However, imaging modalities for lymphatic structure in the abdomen require special instruments, materials, and expertise and are often unreliable [1–7, 12, 13]. Even if preoperative detection of the leakage site is available, the success rate of surgical repair for chylous ascites or abdominal lymphatic fistula has been reported to be low at approximately 50 % because the anatomy of lymphatic systems in the abdomen is inconsistent unlike in the thorax [1–7]. As an initial treatment, therefore, surgical repair is considered to be impractical on many occasions.

As stated above, sporadic case reports showed that TPN with or without octreotide (or somatostatin) is promising for this complication [1–7]. However, these treatments require invasive procedures [8], close medical vigilance, and high medical costs [9–11]. In contrast, oral ED does not require specific instruments or procedures and is available in the outpatient clinic. Elental^®^, which was used in this case, is almost the same formula as VIVONEX^®^ T.E.N. (Nestlé) which is available in many Western countries [14]. Elental^®^ consists of 15 % protein, 83.5 % carbohydrate, and 1.5 % fat (caloric distribution, % of kcal) and can provide nearly fat-free enteral nutrition [15], which does not need nutritional transport via mesenteric lymphatic systems and is therefore reasonable for mitigating chylous ascites [1–7]. Furthermore, Elental^®^ is a completely digested formula and easily absorbed without digestive juice secretion, therefore, seldom causing bowel peristalsis which activates intra-abdominal lymphatic flow [14–16]. From this aspect, Elental^®^ is considered superior to a conventional low-fat diet or medium-chain fat diet which requires digestive juice secretion for absorption and causes bowel peristalsis [1–5, 14–16]. Therefore, Elental^®^ is considered markedly beneficial for decreasing intra-abdominal lymphatic flow and nutritionally promoting wound healing to repair disrupted lymph vessels. It was reported that conservative treatments for symptomatic postoperative chylous ascites required long-term (reportedly 40–90 days) management to achieve complete recovery [1–7]. In our case, this was achieved 28 days after initiating the Elental^®^ diet. Furthermore, the patient was relieved from the severe sense of abdominal fullness shortly after starting the diet. These findings suggest that Elental^®^ may be promising for treating postoperative chylous ascites. When prescribing an oral ED, low compliance was a problem because of its tastelessness [16]; however, various flavors have become available recently and compliance was reported to have increased [16]. If oral application is unendurable, feeding via enteral tube is available and considered to be superior in intrinsic safety to TPN. Thus, fat-free ED can be recommended as an initial treatment for chylous ascites occurring after abdominal surgery because it is easy to start, intrinsically safe, and can be performed in the outpatient clinic if oral intake is available. Lacking strong evidence of its effectiveness, however, other treatment modalities such as TPN with or without octreotide must be initiated when ED is ineffective. Furthermore, surgical repair must always be considered; cases have been reported where conservative management did not cure chyloperitoneum and surgical repair was necessary [1–7]. Hence, it should be emphasized that meticulous, tactful, and appropriate management according to the individual case is essential for this rare but significant complication.

In conclusion, an oral fat-free ED can be recommended as an initial treatment for chylous ascites occurring after abdominal surgery because it is easy to start, intrinsically safe, and available in the outpatient clinic.

## Abbreviations

TPN: Total parenteral nutrition
ED: Elemental diet
CT: Computed tomography
